# A Link Between Local Enrichment and Scalar Diversity

**DOI:** 10.3389/fpsyg.2018.02092

**Published:** 2018-11-01

**Authors:** Chao Sun, Ye Tian, Richard Breheny

**Affiliations:** ^1^Leibniz-Centre General Linguistics, Berlin, Germany; ^2^Division of Psychology and Language Sciences, University College London, London, United Kingdom; ^3^Amazon Research, Cambridge, United Kingdom

**Keywords:** scalar implicature, scalar diversity, scale homogeneity, local enrichment, lexical uncertainty

## Abstract

Several recent studies have shown that different scalar terms are liable to give rise to scalar inferences at different rates (Doran et al., 2009, 2012; van Tiel et al., 2016). A number of potential factors have been explored to account for such *Scalar Diversity*. These factors can be seen as methodological in origin, or as motivated by widely discussed analyses of scalar inferences. Such factors allow us to explain some of the variation, but they leave much of it unexplained. In this paper, we explore two new potential factors. One is methodologically motivated, related to the choice of items in previous studies. The second is motivated by theoretical approaches which go beyond the standard Gricean approach to pragmatic effects. In particular, we consider *dual route* theories which allow for scalar inferences to be explained either using ‘global’ pragmatic derivations, like those set out in standard Gricean theory, or using local adjustments to interpretation. We focus on one such theory, based on the Bayesian Rational Speech Act approach (RSA-LU, Bergen et al., 2016). We show that RSA-LU predicts that a scalar term’s liability to certain kinds of local enrichment will explain some Scalar Diversity. In three experiments, we show that both proposed factors are active in the scalar diversity effect. We conclude with a discussion of the grammatical approach to local effects and show that our results provide better evidence for dual route approaches to scalar effects.

## Introduction

### The Scalar Diversity Phenomenon

**FIGURE 1:**
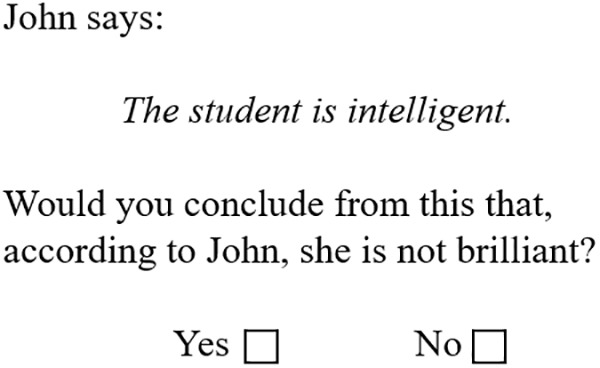
Sample item from van Tiel et al. (2016) – Experiment 2.

Recent experimental studies investigated the rates at which scalar expressions of different lexical categories give rise to scalar inferences (SIs) (Doran et al., 2009, 2012; Beltrama and Xiang, 2012; van Tiel et al., 2016). It has been found in these studies that different scalar expressions give rise to SIs at different rates. van Tiel et al. (2016) employed an inference paradigm to test participants’ interpretation of statements containing scalar expressions. Several classes of scalar expressions were examined including quantifiers (e.g., <all, some>), modals (<certainly, possibly>), adjectives (<beautiful, pretty>) and verbs (<dislike, loathe>). Figure 1 is an example of an item (van Tiel et al., 2016: Experiment 2). Participants read a statement uttered by a character. Then they were asked whether the speaker implied the negation of the stronger statement in which scalar expression was replaced by its stronger scale mate. For example, when the character states that the student is intelligent, participants are asked whether, according to the speaker, the student is not brilliant. A ‘Yes’ response indicates that participants drew the SI and a ‘No’ response indicates that the inference was unavailable.

van Tiel et al. (2016) found significant variation in the derivation rates of SIs across different scalar expressions, ranging from 4 to 100%. Quantifiers and modal expressions generated SIs more frequently than adjectives and verbs. Moreover, while quantifiers and modal expressions consistently gave rise to SIs, there was much greater variability among adjectives and verbs. These results were consistent with those reported in Doran et al. (2009). The scalar diversity effect has been replicated in several studies that have used different procedures and that also provided more context for the target utterance (see Experiment 1 below, also Simons and Warren, 2018; Sun and Breheny, 2018a).

Scalar inference is widely seen as a specific instance of conversational implicature (Horn, 1972; Grice, 1975; Gazdar, 1979; Geurts, 2010). Implicatures are contextual implications of what the speaker literally says, which are derived on the basis of expectations speakers and listeners have about each other. A scalar implicature for the experimental item, ‘The student is intelligent,’ mentioned above, would be that the student is not brilliant. It is widely agreed that the underlying meaning of ‘intelligent’ is such that someone counts as intelligent if their intellectual capacities place them anywhere at or above some standard of such capacities. Another scalar term whose meaning relates to the same scale may be anchored to a higher point. This would be the case with ‘brilliant.’ Thus the student being brilliant is consistent with a literal assertion of ‘The student is intelligent.’ The standard Gricean explanation for the SI, *the student is not brilliant* is based on the idea that interlocutors expect each other to be as informative, or specific as is relevant in context (see for example Geurts, 2010). From this expectation, one can reason to the conclusion that, according to a speaker who used ‘intelligent,’ they do not consider the student brilliant. According to the design of van Tiel et al.’s (2016) study, all of the pairs of scalar terms have literal meanings with this scalar property. That is, the term that is not mentioned picks out a higher point on a scale than the one that is mentioned in the speaker’s utterance. Thus, for all of the items used, the standard approach implies that a SI could be available. Although this standard account does not predict that there should be no scalar diversity, it does not predict that there will be diversity; at least not without further assumptions. For instance, there could be differences in terms of the relation between the weaker term uttered and the stronger alternative that needs to be evoked in order to derive the implication. Thus the interest in the scalar diversity phenomenon surrounds the question of what would explain this great variation in rates of ‘Yes’ response for different scalar terms.

In this paper, we will approach our discussion of factors responsible for scalar diversity in terms of their being either methodologically or theoretically motivated. Among theoretically motivated factors, we consider factors suggested by the standard Gricean theory and those that would follow from an augmented standard theory, which accommodates the widely acknowledge fact of local pragmatic effects.

In the following sub-section, we review empirical work so far presented that has accounted for some of the scalar diversity effect. Here, we introduce a new methodological factor, related to the polysemy of many of the scalar terms. We then introduce the idea that certain ‘local’ effects are not explained by the standard Gricean mechanism. We discuss an account of this phenomenon within the Bayesian, Rational Speech Act framework. We show how this RSA framework predicts scalar diversity to the extent that scalar terms are susceptible to local pragmatic processes.

In the second part of the paper, we present three experiments. The first is a replication of van Tiel et al.’s (2016) study. The second addresses the methodological problem of polysemy of scalar terms. The third tests the prediction concerning the relation between local enrichability and scalar diversity.

### Accounting for Scalar Diversity

If we approach the results of van Tiel et al.’s (2016) study from the standard Gricean perspective, one potential factor that may contribute to the scalar diversity effect is the lack of context in the experimental items. If we reconsider the item in Figure 1 above, we can see that the utterance is presented without context. It is widely agreed that, from a Gricean perspective, stronger alternatives should only be considered for SI if that alternative is somehow relevant in context. Several experimental studies have shown that participants are able to infer implicit relevant context with the presentation of an experimental stimulus (Breheny et al., 2006; Bergen and Grodner, 2012). While all the scalar items are tested in van Tiel et al.’s (2016) study without context, it could be that items differ in the extent to which the relevant context can be inferred for different scalar terms. van Tiel et al. (2016) consider this possibility and dismiss it as likely to be an explanation for Scalar Diversity. Their case is supported to some extent by evidence from Doran et al. (2009). In that study, the sentence containing a scalar term is presented in explicit contexts that make the more informative alternative relevant and in explicit contexts that do not. Doran et al. (2009) report that rates of SI are affected by this contextual manipulation for their adjective scales but not for their quantifier scales (e.g., involving ‘some’). But even in supportive context, Doran et al. (2009) found that rates for quantifiers were higher than for adjectives. Thus, the presence of explicit supportive context lessens the difference between scale types, it does not eliminate it. Further support for this conclusion comes from a corpus study reported in Sun and Breheny (2018a). Here participants read items selected from a corpus that had a wide variety of contexts. Again, the scalar diversity effect was lessened by the richer contexts associated with the items, but not eliminated.

van Tiel et al. (2016) explored a range of other explanations for the variability which they found. These explanations are motivated by standard approaches to SI since they focus on the relation between the scalar term used and its alternative. van Tiel et al. (2016) hypothesized that the availability of the stronger alternative and the distinctness of the scale-mate may account for some of the variability in inference rates. The availability of the stronger alternative was measured in four parameters including association strength, grammatical class (open/closed), semantic relatedness and relative frequency of the scale-mate. One motivation for exploring availability might be that pairs of scalar terms may be more or less strongly associated with one another and this might be a factor in Scalar Diversity. However, in a regression analysis, van Tiel et al. (2016) found that none of the four parameters related to availability could independently explain scalar variability. This finding is corroborated in the study reported below, and in Sun and Breheny (2018a). A caveat should be entered at this point regarding measures of association. These have all been tested against the result of studies like van Tiel et al.’s (2016) inference task where the task stimuli mention the stronger scalar term as well as the weaker one. That is, in Figure 1 above, ‘brilliant’ is mentioned as well as ‘intelligent’; ‘all’ is mentioned as well as ‘some’; and so forth. By mentioning the stronger scalar term (‘brilliant,’ ‘all,’ etc.) the task design may neutralize any difference in salience that might antecedently exist among scalar pairs. Thus it is possible that differences in association among scalar pairs could contribute to the scalar diversity effect, but that would be on top of other factors at play in the results reported to date.

The second kind of factor that van Tiel et al. (2016) consider is the distinctness of the scale-mate. Specifically, they sought a measure semantic distance (i.e., the difference in the perceived strengths between the pairs of scalar terms) and ‘boundedness’ (i.e., whether the underlying scale contained an endpoint). In contrast to measures of association, a regression analysis showed that semantic distance and boundedness did independently account for a significant amount of variance, where boundedness accounted for over three times more variance than did semantic distance.

Together, all of the measures explored by van Tiel et al. (2016) accounted for less than half of the variance, leaving a large amount of variation unexplained. Factors to do with the relation between scalar term and its alternative are the ones that are clearly suggested by the standard Gricean approach to SI. van Tiel et al. (2016) suggest that the availability of the stronger alternative and the distinctness of scale-mate are the only plausible factors that they could think of. Their conclusion is that the rest of the variation in inference rates among scalar terms must be unsystematic. In the rest of this section, we discuss two other kinds of factor motivated by considerations beyond standard Gricean theory.

#### Methodological Factors

The first thing to consider about the scalar diversity effect is that there might be factors related to the methods used in these studies that contribute to the effect. One such factor is identified in Benz et al. (2017). This relates to the phenomenon known as negative strengthening. A negated scalar term might not simply denote the complement of its positive counterpart but may be understood with a strengthened meaning. For example, ‘not tall’ is often understood not simply as denoting the set of things that are not at or above the contextual reference point in height, but somewhat below this standard. Negative strengthening is relevant to the methods used in van Tiel et al. (2016). Consider for example the item in Figure 1. The participant is asked to judge if the speaker thinks the student is not brilliant. To the extent that the scalar term under negation may undergo negative strengthening, the participant may respond negatively on the basis that ‘not brilliant’ is understood to mean somewhat less than brilliant, e.g., stupid. Benz et al. (2017) provide some evidence that adjective terms are more susceptible to negative strengthening and so this may have been a factor in van Tiel et al.’s (2016) results. However, it is not likely to be the sole remaining factor since other studies have probed for SIs without this kind of stimulus and still found the scalar diversity effect. For example, Sun and Breheny (2018a) employ the paraphrase task from Degen (2015). This task asked the participant whether ‘intelligent but not brilliant’ would be a good paraphrase for ‘intelligent’ in a given item. Here, there is no conflict with a negative strengthening inference.

In this paper, we wish to explore an issue related to items used in van Tiel et al.’s (2016) studies and others. This has to do with how homogeneous the senses of the scalar terms are. The relevant concept here is that scalar terms, such as ‘brilliant’ can be highly polysemous. ‘Brilliant’ can be understood as related to an underlying intelligence scale, but it can also be understood to be related to other scales to do with personality, such as kindness, or with other skills, as in a brilliant actor. Consider also the scale <unsolvable, hard> taken from van Tiel et al. (2016). ‘hard’ has a sense related to difficult. Under this sense, ‘unsolvable’ could be the hyponym of ‘hard’ with respect to problem-solving (e.g., ‘this is a really hard question’), while ‘unbearable’ could be the hyponym of ‘hard’ with respect to suffering (e.g., ‘times were hard at the end of the war’). Thus, it is sometimes the case that ‘unsolvable’ is not construed as being on the same entailment scale as ‘hard,’ and the same happens with other scales such as <depleted, low>, <ridiculous, silly>, and <happy, content>.

When asked to judge whether ‘hard’ implies ‘not unsolvable’ or whether ‘low’ implies ‘not depleted,’ participants in van Tiel et al.’s (2016) experiments may have evoked senses of these terms that are not on the same scale. By contrast, consider the scale <always, sometimes>, ‘sometimes’ and ‘always’ have fairly homogeneous senses across uses, relating to the frequency of an event. It would be difficult to construe these terms as not being in an entailment relation. Thus, when asked to judge whether ‘sometimes’ implies not always, participants were more likely to derive an implicature. We hypothesize that other things being equal, the more homogeneous the sense of the items in a pair, the higher the rate of scalar implicature derivation. We will test this hypothesis in Experiment 2.

#### Theoretically Motivated Factors

Beyond methodological questions, we want to consider whether scalar diversity can be explained to some extent if we consider pragmatic theories that go beyond standard Gricean theory. In particular, standard Gricean theory has long been the target of criticism that the method of deriving conversational implicatures cannot explain a large class of apparently pragmatic effects (Cohen, 1971; Wilson, 1975; Carston, 1988). This critical work shows that in some cases, the meaning of a sub-constituent of an utterance seems to be given a pragmatically augmented interpretation. Although early work on such ‘local enrichment’ did not focus on SI, recent research has (Noveck and Sperber, 2007; Chierchia et al., 2012; among others). An example of local enrichment involving SI is given in (1a) below, which could be glossed by imagining the constituent ‘hit some of the targets’ being given a reading, *hit some and not all of the targets*. This is indicated in (1b):

1. a. Exactly one player hit some of the targets.
b. Exactly one player hit some but not all of the targets.

This example is based on materials in Chemla and Spector (2011) who discuss why the gloss in (1b) is not derivable using standard Gricean derivation. Potts et al. (2016) reports that participants in an experiment readily understood sentence (1a) according to the gloss in (1b). That local enrichment does occur in natural language is becoming a more widely accepted assumption.^1^ Although very little experimental research has explored the conditions under which local processes occur, it is possible to incorporate the fact of local enrichment into a framework that also allows for ‘global’ implicature derivation, of the kind set out in the standard Gricean theory.^2^ Such a dual-route framework is set out in Bergen et al. (2016) which augments a ‘standard’ Bayesian probabilistic approach to scalars, the Rational Speech Act (RSA) approach, with additional ‘lexical uncertainty’ (RSA-LU). This framework adopts a liberal stance toward (local) enrichment and posits a family of compositional semantic rules, each of which can represent different enrichments of a given constituent. This is coupled with a framework for reasoning with the uncertainty about which, if any, enrichment is being used. In order to see how such a dual-route approach might account for scalar diversity, it will be necessary to briefly outline some of the details of RSA-LU.^3^

The RSA approach aims to capture how speakers and listeners recursively model each other’s production and comprehension decisions. Like the standard Gricean approach, the standard RSA approach to SI assumes that a single literal interpretation could be assigned to a sentence containing a scalar term. A ‘literal listener’ uses Bayesian inference to model a speaker who chooses an utterance, *u*, on the assumption that (the speaker believes) it is true. If we assume that a literal interpretation of the sentence uttered determines the function ℒ from utterances and states of affairs to truth values, then the probability that the literal listener assigns to each state of affair after hearing the utterance, *L*_0_, is determined by the prior probability on the state of affairs and the truth value of utterance in that state of affairs as follows:

2. $L_{0}(w|u)\;\propto \;P(w)L(u,\;w)$

A pragmatically sophisticated speaker who addresses *L*_0_ intending convey what is the case, is best served by choosing an utterance that is maximally specific, subject to preferences related to cost of the message. Putting aside some details, the distribution for the speaker’s choice of utterance is given as in (3-4) below:

3. $S_{1}(u|w)\;\propto \;e^{\lambda U_{1}(u|w)}$
4. $U_{1}(u|w)=\log(L_{0}(w|u))-C(u)$

Then a pragmatically sophisticated listener may make inferences about S_1_’s message according to Bayes’ rule:

5. $L_{1}(w|u)\;\propto \;P(w)S_{1}(u|w)$

Higher-order iterations, *S*_n_ and *L*_n_, follow the same pattern.

This standard RSA model is capable of accounting for the fact that if the speaker says, ‘The nurse saw some of the signs,’ we are liable to infer that (according to the speaker) the nurse did not see all of the signs. In general, for scalar pair <S, W>, where S is stronger than W, if the speaker utters W, we are liable to infer that she does not think S is true (see Bergen et al., 2016 for an illustration). Thus using only a single ‘literal’ semantic interpretation function, RSA shows that Bayesian reasoning among speaker and hearer can result in a SI. This in essence provides an account of SI in a broadly ‘Gricean’ way.

However, as mentioned, one can factor in the possibility of enrichments that cannot be explained using a ‘global’ Gricean inference which assume the literal semantics of the sentence. Thus, Bergen et al. (2016) allow that the speaker may use, and be understood to be using, an enriched interpretation of a certain clause type, or expression type. This can be done by supposing that each kind of enrichment for W constitutes a new semantic interpretation function *L*_i_. Uncertainty about which, if any, enrichment is being employed in a given utterance can be captured at the level of the first pragmatically sophisticated listener, *L*_1_, who marginalizes (takes the weighted average) over interpretation functions relative to the prior probabilities of each possible enrichment being used. This is indicated in a revised set of formulae in (6–9) below:

6. $L_{0}(w|u,\;L)\;\propto \;P(w)L(u,\;w)$
7. $S_{1}(u|w,\;L)\;\propto \;e^{\lambda U_{1}(u|w)}$
8. $U_{1}(u|w,\;L)=\log(L_{0}(w|u,\;L)-C(u)$
9. $L_{1}(w|u)\;\propto \;P(w)\;\sum_{L\in \wedge }P(L)S_{1}(u|w,\;L)$

The upshot of this move for simple cases containing unembedded scalar terms is that the strength of the SI (that the speaker does not believe that the stronger sentence is true) can be affected by the prior probability that the speaker intends the literal interpretation or one of the possible enrichments of W. If there is a high prior probability that the scalar term’s interpretation gets locally enriched to exclude states of affairs where S is true, then, overall, the strength of the SI that S is not true would be greater than it would be if no enrichment were used (i.e., if only the standard model were used). Thus, if the scalar term W is associated with a very low, or zero, prior probability that it is enriched this way, then the strength of the SI in a stimulus like that presented in van Tiel et al. (2016) will be lower than where it has a higher prior probability of such local enrichment.^4^

Let us refer to an enrichment of the interpretation of W so that it excludes cases where S is true as *upper-bound excluded local enrichment (UBELE)*. It is in principle possible that scalar terms differ in the prior probabilities on this kind of enrichment. To the extent that these priors differ across scalar terms, we should see differences in rates of SIs in the task reported in van Tiel et al. (2016). Thus, RSA-LU predicts that variation in the strength of these priors could explain at least some of the scalar diversity effect. We explore this prediction in Experiment 3.

## The Current Studies

We tested three separate groups of people in Experiments 1–3. Experiment 1 is more or less a replication of Experiment 2 of van Tiel et al. (2016) using a different measurement scale. Our goal is to obtain a continuous measure of participants’ judgment on the availability of SIs for each scalar pair. The remaining studies investigate whether scale homogeneity or liability of UBELE can account for some of the variation in the rates of SIs.

Scale homogeneity was operationalized in terms of a naturalness judgment on an ‘X but not Y’ construction where <X, Y> is a scalar pair and X can be understood as stronger than Y. In Experiment 2, a group of participants was asked to rate the naturalness of sentences of the form ‘X but not Y,’ e.g., (10a–c):

10. a. The student is brilliant but not intelligent. <brilliant, intelligent>
b. The water is hot but not warm. <hot, warm>
c. The dancer finished but she did not start. <finish, start>

‘But’ has a *denial-of-expectation* conventional implicature. Thus, a sentence ‘X but not Y’ is felicitous to the extent that X can be construed to not strictly entail Y, but Y would normally be expected, given X. A scale with high homogeneity is one where the stronger term is interpreted to entail the weaker term. Entailment relations require that if X entails Y, whenever X holds, Y must hold. Therefore these ‘X but not Y’ sentences should be very unnatural if the contrasting predicates X and Y are on the same entailment scale. So if the naturalness rating for a ‘but’ sentence is low, it suggests a high degree of homogeneity for the given scale; whereas if the rating is high, then the degree of homogeneity is relatively low. Other things being equal, the more homogeneous the sense of the items in a pair, the higher the rate of scalar implicature derivation. We predict that the naturalness rating for scalar expressions in Experiment 2 should negatively correlate with the results of Experiment 1.

Liability of UBELE is the degree to which a weak scalar term is liable to undergo local enrichment to exclude states of affairs where the stronger term is true. In Experiment 3, liability of UBELE is operationalized in terms of the naturalness judgment of an ‘X so not Y’ construction where <X, Y> is a scalar pair and X is stronger than Y. In Experiment 3, a separate group of participants rated the naturalness for sentences of the form, ‘X so not Y,’ e.g., (11a–c).

11. a. The student is brilliant so not intelligent. <brilliant, intelligent>
b. The water is hot so not warm.<hot, warm>
c. The dancer finished so she did not start. <finish, start>

The discourse function of ‘so’ contrasts with that of ‘but’ in a number of ways (Blakemore, 2002). ‘So’ implies that the second segment follows in some way from the first. While ‘X but not Y’ suggest that one might expect Y, given X, ‘X so not Y’ suggests that one might expect not Y, given X. Thus, ‘X so not Y’ sentences should be more coherent to the extent that the weaker scalar expression can undergo UBELE. For example, to understand (11b) as felicitous, ‘warm’ must have its meaning locally enriched to be understood as ‘warm but not hot.’ Notice that this has to involve local enrichment rather than Gricean scalar-implicature reasoning because the weaker term is in the scope of negation.^5^

In Experiment 3, if the naturalness rating for ‘so’ sentences is low, it suggests that the scalar expression is less liable to be enriched to exclude the upper bound; whereas if the rating is high, then it is more liable to be so enriched. As mentioned above, RSA-LU predicts that greater liability for UBELE, the higher the ratings in an inference task of the kind presented in van Tiel et al. (2016). Thus, we predicted that the naturalness rating for scalar expressions in Experiment 3 should positively correlate with the results of Experiment 1.

## Experiment 1

### Methods

#### Participants

Thirty-six participants were recruited from University College London via an online psychological subject pool. All participants spoke English as a native language. Participants provided written informed consent, and this study was approved by the UCL Research Ethics Committee. Participants came into the lab to complete the testing on a laptop, in return for course credit or £2.5.

#### Materials and Procedure

We tested all 43 scale pairs from van Tiel et al. (2016) in an inference task to measure scalar implicature derivation. The only difference in procedure was that, instead of providing a yes/no response, participants were asked to rate on a 0–100 scale to indicate to what extent they could infer from the speaker’s statement that the speaker does not believe the stronger alternative. In van Tiel et al. (2016) Experiment 2, the statements were created based on the results of the sentence completion task, e.g., ‘The __ is attractive but she isn’t stunning.’ Three statements were selected for each scale, partially based on the completion frequency. Here, we selected the two more frequent statements for every scale (see Appendix for a list of items used). If the statements used in the original study had the same completion frequency, a random selection was made. We also used the exact same control items from van Tiel et al.’s (2016) experiment. Four lists were created, each participant judged either 21 or 22 experimental items and 7 control items. Thus, each experimental item was judged by 18 participants. Participants were randomly assigned to one of four lists. A randomized order of presentation of the items was created for each participant.

### Results

**FIGURE 2:**
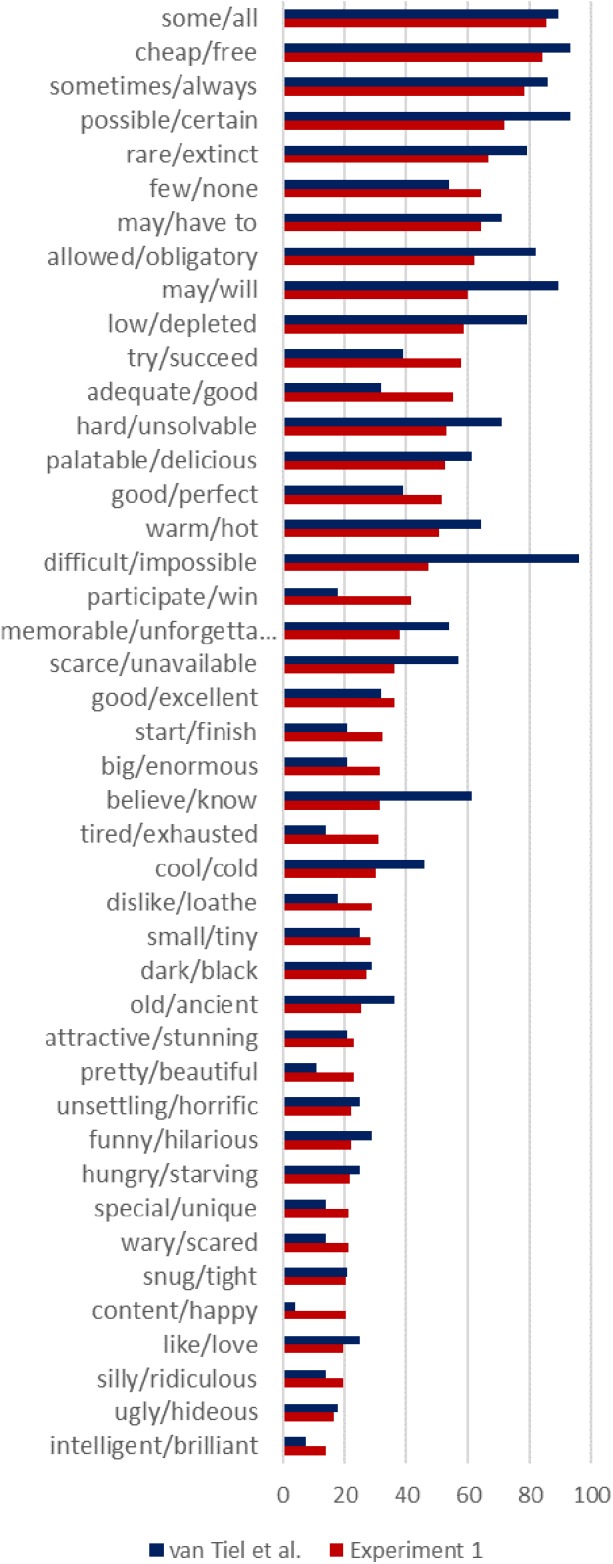
Mean inference ratings for Experiment 1. The rates of SIs from van Tiel et al. (2016) Experiment 2 are shown in blue bars.

The mean ratings for entailments and non-coherent inferences were 86.97 (*SD* = 24.81) and 8.3 (*SD* = 15.09), respectively. Two participants were excluded from the analysis because their mean ratings for entailments or non-coherent inferences were two standard deviations away from the means. The mean ratings for all scalar items are shown in Figure 2 (red bars). The rates of SIs from van Tiel et al. (2016, Experiment 2) are also included in that figure (blue bars).^6^

We carried out one-way ANOVAs with the ratings on the inference task as the dependent variable and lexical categories as the independent variable. The ratings were averaged by items (43 scales) before entering into the analysis. There was a statistically significant difference among lexical categories [*F*(3,39) = 9.52, *p* < 0.001]. A Tukey *post hoc* test revealed that the ratings of SI for quantifiers (*M* = 76.03, *SD* = 10.89) and modals (*M* = 64.35, *SD* = 5.24) were significantly higher than for adjectives (*M* = 34.95, *SD* = 17.19) and verbs (*M* = 35.30, *SD* = 13.17), but there was no statistically significant differences between quantifiers and modals, and between adjectives and verbs. These results are in line with those seen in van Tiel et al. (2016). Inspecting the graph, one can see some differences among items, but the general pattern is the same.

To examine whether factors identified by van Tiel et al. (2016) explain some of the variation found in Experiment 1, we conducted a multiple linear regression analysis to predict the ratings of SIs in our Experiment 1 from all the potential factors reported in van Tiel et al. (2016) including association strength, grammatical class, word frequencies, semantic relatedness, semantic distance, and boundedness. The ratings of SIs in Experiment 1 were averaged by item (43 scales) before entering the analysis. The results of the linear regression are summarized in Table 1. The model explained 48.7% of the variance [*R*^2^= 0.56, *F*(6,35) = 7.48, *p* < 0.001]. As in van Tiel et al. (2016) only semantic distance and boundedness were significant predictors of the inference task results, whereas other factors did not make a significant contribution to the model.

**Table 1:** Results of multiple linear regression for inference ratings of Experiment 1.

	Estimate	*SE*	*t*-Value	*p*-Value	*R*^2^
(Intercept)	4.651	21.135	0.22	0.827	
Association strength	0.024	0.108	0.22	0.827	0.007
Grammatical class	-13.575	9.429	-1.44	0.159	0.099
Word frequencies	-3.603	2.605	-1.38	0.175	0.016
Semantic relatedness	3.036	14.085	0.22	0.831	0.020
Semantic distance	7.234	3.203	2.26	0.030	0.106
Boundedness	20.802	4.897	4.25	0.000	0.315


### Discussion

Experiment 1 established that there is a considerable amount of variation among scalar terms in terms of how strongly they give rise to scalar implicatures. The general pattern found in van Tiel et al. (2016) was replicated, with a different measurement scale. Experiment 1 also replicated van Tiel et al.’s (2016) findings that semantic distance and boundedness only explain some of the variation.

## Experiment 2

### Methods

#### Participants

We invited Amazon Mechanical Turk workers located in the United States with a 95% approval rate on tasks previously performed for other requesters. Forty participants were recruited and were paid United States $0.50 for their participation. The experiment was initiated by a consent statement approved by UCL Research Ethics Committee. Participants were asked to indicate their native language, but we paid them regardless of their answer to this question. Only participants with English as a native language were included in the analysis.

#### Materials and Procedure

**FIGURE 3:**
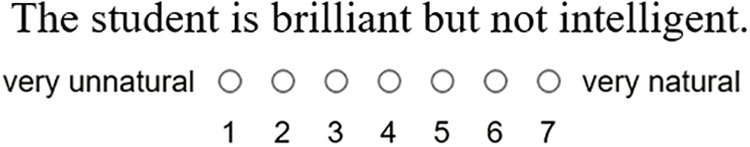
Sample item in Experiment 2.

Figure 3 is an example item. We used the 43 scales investigated in Experiment 1 to construct experimental sentences for Experiment 2. The experimental sentences were of the form ‘X but not Y’ where, according to van Tiel et al. (2016), X and Y are a pair of scalar terms and X is stronger than Y. For example, ‘The student is brilliant but not intelligent.’ We constructed two experimental sentences for every scale (see Appendix for a list of items used). The nominal (‘student’) used in each experimental sentence was the same as for the corresponding statement in Experiment 1. For the verbs and auxiliary verbs like ‘may,’ experimental sentences were constructed differently to make sure that the weaker term was in the scope of negation (see Appendix for details); for instance, ‘The lawyer will appear in person but it is not the case that he may appear in person.’ In addition, we constructed seven filler sentences, which contained clearly felicitous (e.g., ‘The banker is rich but not happy’) and clearly infelicitous items (e.g., ‘The man left the party but he never came’). Participants were asked to rate how natural these constructions are on a 1 (very unnatural) – 7(very natural) scale. Each participant judged 43 experimental sentences and 7 fillers. Eight survey versions with pseudo-randomized order of items were created. Participants were randomly assigned to one of eight surveys.

### Results

**FIGURE 4:**
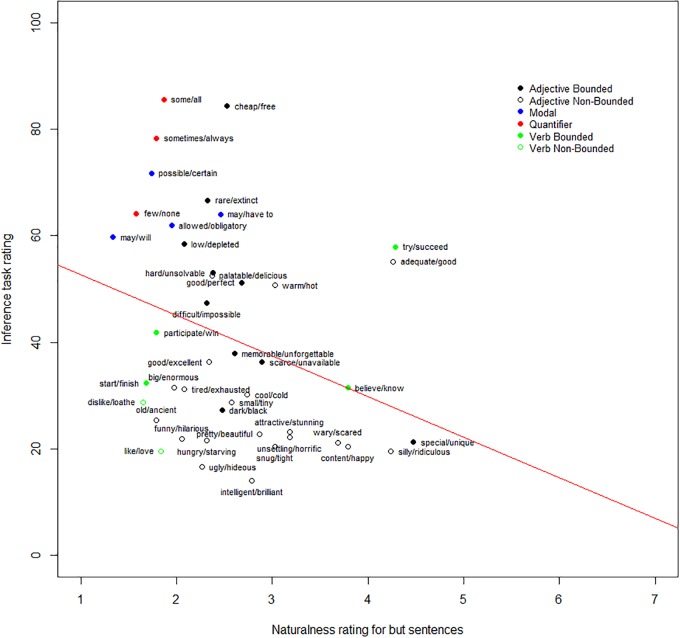
Negative correlation between the absence of homogeneity and inference rate.

Two participants were excluded because their mean ratings for the infelicitous items were above 5. The mean ratings for the clearly felicitous and clearly infelicitous control items were 5.8 (*SD* = 1.82) and 2.32 (*SD* = 1.91). The mean rating for experimental items ranged from 1.33 (*SD* = 0.59) (<will, may>) to 4.47 (*SD* = 2.11) (<unique, special>). Critically, we found that the naturalness of the ‘but’ sentences correlated negatively with the ratings of SIs in Experiment 1 [*r*(41) = -0.31, *p* = 0.04] – see Figure 4. In addition, it correlated negatively with the results from van Tiel et al. (2016, Experiment 2) [*r*(41) = -0.36, *p* = 0.02]. These results confirmed the prediction outlined earlier. We defer discussion of these results until after the combined analysis.

## Experiment 3

### Methods

#### Participants

Forty participants were recruited from University College London via an online psychological subject pool. All participants spoke English as a native language. Participants provided written informed consent, and this study was approved by the UCL Research Ethics Committee. Participants came into the lab to fill out a paper-based survey, in return for course credit or £1.

#### Materials and Procedure

**FIGURE 5:**
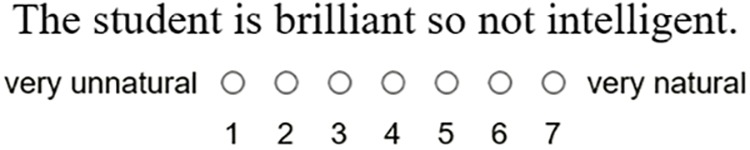
Sample item in Experiment 3.

Figure 5 is an example item. We used 43 scales investigated in Experiment 1 to construct experimental sentences for Experiment 3. Two experimental sentences were constructed for each scale (see Appendix for a list of items used). The experimental sentences were of the form ‘X so not Y,’ where X is stronger than Y; for example, ‘The student is brilliant so not intelligent.’ As in Experiment 2, the nominal (‘student’) used in each experimental sentence was from statements used in Experiment 1. For the verbs and auxiliary verbs like ‘may,’ experimental sentences were constructed differently (see Appendix for details); for example, ‘The lawyer will appear in person so it is not the case that he may appear in person.’ Seven filler sentences were constructed, which contained clearly felicitous (e.g., ‘The cup is red so not blue’) and clearly infelicitous items (e.g., ‘The banker is rich so not happy’). Participants were asked to indicate how natural these constructions are on a 1 (very unnatural) – 7 (very natural) point scale. Each participant judged 43 experimental sentences and 7 fillers. Eight paper-based survey versions with pseudo-randomized order of items were created.

### Results

**FIGURE 6:**
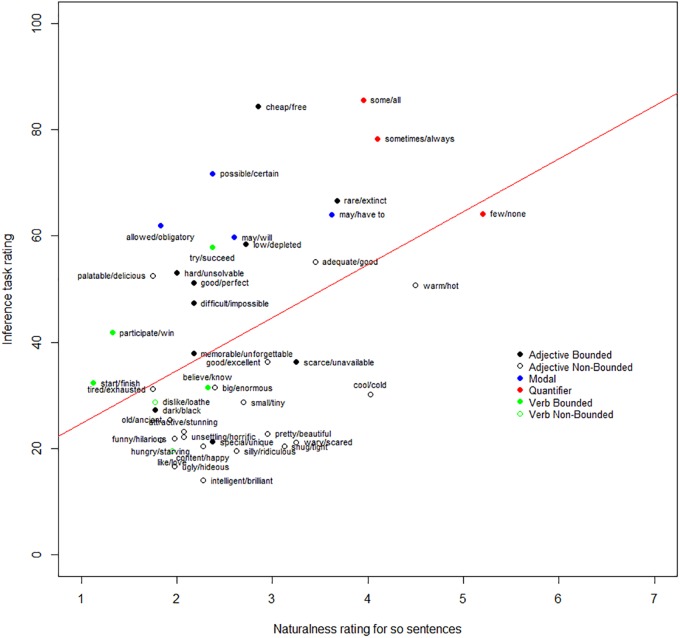
Positive correlation between the propensity of local enrichment and inference rate.

The mean rating for the clearly felicitous and clearly infelicitous control items were 5.89 (*SD* = 1.68) and 1.53 (*SD* = 1.25). The mean rating for experimental items ranged from 1.13 (*SD* = 0.33) (<finish, start>) to 5.2 (*SD* = 1.99) (<none, few>). We found that the naturalness of the ‘so’ sentences positively correlated with the ratings of SIs in Experiment 1 [*r*(41) = 0.44, *p* = 0.004] – see in Figure 6. In addition, the naturalness of the ’so’ sentence also positively correlated with the results from van Tiel et al. (2016, Experiment 2) [*r*(41) = 0.35, *p* = 0.02].

## Combined Analysis

To investigate the proportion of variance explained by all the potential factors, multiple linear regression analyses were conducted to predict the ratings of SIs in Experiment 1 from scale homogeneity degree, propensity for local enrichment, and all factors established in van Tiel et al. (2016). The rating of SIs in Experiment 1, and the naturalness rating from Experiments 2 and 3 were averaged by item (43 scales) before entering the analysis. The results of the linear regression are summarized in Table 2.

**Table 2:** Results of combined analysis.

	Estimate	*SE*	*t*-Value	*p*-Value	*R*^2^
(Intercept)	-31.274	24.967	-1.250	0.219	
Scale homogeneity	-3.142	3.008	-1.040	0.304	0.037
Local enrichment	10.442	2.668	3.910	<0.001	0.149
Association strength	0.048	0.092	0.520	0.606	0.006
Grammatical class	1.506	9.032	0.170	0.869	0.067
Word frequencies	-2.926	2.262	-1.290	0.205	0.013
Semantic relatedness	-8.151	12.303	-0.660	0.512	0.015
Semantic distance	8.291	3.150	2.630	0.013	0.107
Boundedness	21.564	4.171	5.170	<0.001	0.308


We found that the regression model accounted for 63% of the variance [*R*^2^ = 0.70, *F*(8,33) = 9.73, *p* < 0.001]. This contrasts with the 49% of variance explained without the ratings for the ‘but’ and ‘so’ tasks entered in the model. In this fuller model, the propensity for local enrichment, semantic distance and boundedness were substantial factors, with the propensity for local enrichment explaining 15%, semantic distance explaining 11%, and boundedness explaining 31%. None of the other factors significantly accounted for the variation in the rates of SIs. In this model, scale homogeneity did not significantly explain the variance. Scale homogeneity was highly correlated with semantic distance [*r*(41) = -0.53, *p* < 0.001]. Thus, the variance in inference ratings explained by scale homogeneity largely overlapped with the variance accounted for by semantic distance. When semantic distance was omitted from the model, scale homogeneity did explain a significant amount of the variance.

## General Discussion

### Discussion of Experiments 2 and 3

We adapted the items from van Tiel et al. (2016, Experiment 2) for these two naturalness judgment tasks. Participants were asked to judge the felicity of sentences of the form ‘S but/so not W’ where ‘S’ is the stronger term from the SI judgment task (‘all,’ ‘hot,’ etc.) and ‘W’ is the weaker term (‘some,’ ‘warm,’ etc.). The respective sentences have different felicity conditions due to the function of ‘but’ and ‘so,’ respectively. We argue that the ‘but’ sentences probe scale homogeneity, while the ‘so’ sentences probe liability for UBELE.

Concerning scalar homogeneity, if participants find a way to read a sentence of the form ‘S but not W’ as felicitous, then it indicates that the items of this scalar pair could be constructed as not always being on the same scale. The results of Experiment 2 showed that the degree of homogeneity varied across different scales. That is, quantificational and modal scales, as well as most verb scales, are in clear entailment relation, but most adjective scales are not. We suggest that this variation in the degree of homogeneity is expected due to factors like underspecification or polysemy. We found that high homogeneity led to higher rates of SIs, compared to when homogeneity was low.

The results of Experiment 2 are related to the hypothesis discussed in Doran et al. (2009). They suggested that there are domain-general scalar expressions such as quantifiers and modals and domain-specific ones such as adjectives. The former are more likely to give rise to SIs in the absence of context, whereas the latter require more contexts in order to derive SIs. Doran et al. (2009) found that only the derivation of adjective scales was affected by providing stronger scale mates in the context. This result might be due to the low homogeneity in adjective scales. That is, without restriction in the context, the use of scalar adjectives may evoke alternatives that are irrelevant in deriving scalar implicatures.

Since scalar homogeneity is strongly correlated with semantic distance, it raises the question of what the relation between the two concepts is. One possibility is that a low rating of semantic distance reflects the fact that scalar pairs are not uniformly on the same scale. That is, as it was measured in van Tiel et al. (2016), semantic distance may reflect, to some extent, both genuine semantic distance (a measure of distinctness) and scale homogeneity. For example, a high semantic distance rating for <all, some> may reflect genuine distinctness of the terms, while a lower rating for <unique, special> may reflect also a lack of scale homogeneity. Future research may seek an alternative means to measure distance that may de-confound these two dimensions.

Turning now to liability for UBELE, this is a new factor motivated by an extension of standard Gricean pragmatic theory. In Experiment 3, if participants find a way to read the sentences of the form ‘S so not W’ as felicitous, then it indicates that ‘W’ (e.g., a sentence containing ‘some’) has been locally enriched in the scope of negation to exclude situations where S is true (e.g., all). The results of Experiment 3 showed that the naturalness of ‘S so not W’ varied across different scales, suggesting that scalar terms differ in their propensity for being locally enriched in this way. The very strong positive correlation between the naturalness of ‘S so not W’ and the rates of SIs measured in the inference task suggested that liability of UBELE influences the judgment in van Tiel et al.’s (2016) original inference task. UBELE can give rise to what looks like a standard Gricean scalar implicature in the unembedded case and this could have inflated rates measured in the inference task.

### Theoretical Implications of Scalar Diversity

From the perspective of the standard Gricean approach to SIs, the existence of a scalar diversity effect among apparently good scalar pairs is not predicted without further assumptions. In previous research, a number of factors have been explored to account for scalar diversity. Apart from one methodologically motivated factor, these factors can all find motivation from the perspective of standard Gricean approaches to scales, relying on scalar alternatives. To date some variance has been explained by these theoretically motivated factors but much is left unexplained. Our contribution in this paper has been to add one more potential methodological factor (scale homogeneity) and one more theoretically motivated factor (liability to upper-bound excluding local enrichment).

As to scale homogeneity, we obtained the predicted negative correlation between ratings on our ‘but’ task and those on the inference task. However, these ratings were highly correlated with ratings for semantic distances and, in a full model that also includes semantic distance as a factor, ‘but’ task ratings did not emerge as a significant factor. We have indicated how future research may explore to what extent it is lack of semantic distance and lack of scale homogeneity explain low rates of implicature, particularly for adjective items.

The results of the ‘so’ task clearly suggests a new factor unexplored in previous studies. This task operationalizes our idea that weak scalar terms differ in their propensity for being locally enriched to exclude the upper bound (UBELE). Our results provide confirmation for current pragmatic approaches to scalars that extend the standard Gricean approach to accommodate the fact of local enrichment. We focused in particular on RSA-LU (Bergen et al., 2016), to derive a prediction of a positive correlation between ratings on our ‘so’ task and the inference task. This is what we found. Moreover, we established that a model including this measure of liability for UBELE as a factor accounts for more variance than a model which includes only those factors motivated by the standard Gricean approach, explored in van Tiel et al. (2016).

We note, however, that ‘distinctiveness of alternatives’ factors, motivated by the standard Gricean model, remained significant in accounting for scalar diversity. This is expected in a dual-route pragmatic approach like RSA-LU. For in that approach, there are two routes to SI. One route is via so-called, ‘global’ inference about the speaker’s actions employing the literal semantics of the sentence and shared principles of conversation. This is akin to the standard Gricean derivation which relies on scalar alternatives. Thus distinctness of those alternatives, as well as their contextual relevance and availability, remain potential factors. The other is via a free enrichment process. That factors motivated by both routes contribute to accounting for Scalar Diversity is expected on the dual route account.

Until now we have not discussed grammatical theories of scalar implicature phenomena. According to widely cited versions of these theories (e.g., Fox, 2007; Chierchia et al., 2012), scalar implicatures of the kind tested in van Tiel et al.’s (2016) inference task are not derived using general pragmatic principles but result from the presence of an exhaustification operator in the syntactic representation of the sentence. This operator functions like ‘only’ in two important respects; (i) it may be placed at different scope sites within a sentence; (ii) in all cases it is interpreted relative to alternatives to its argument. To illustrate this point, for (1) the exhaustification operator would be represented as taking only a constituent, ‘x hit some of the targets’ in its scope, leading to alternatives like, ‘x his all of the targets.’ For sentences where there is apparently a ‘global’ SI, like the items in our Experiment 1, the operator takes scope over the whole sentence. For example, when participants infer that ‘The student is intelligent’ implies she is not brilliant, this would be explained in terms of an operation on the whole sentence, with ‘The student is brilliant’ as alternative. Thus there are two key differences to dual route theories described above. The first is that the grammatical approach posits only a *single* mechanism to account for both local effects of the kind involved in Experiment 3 and ‘global’ effects tested in the inference task, Experiment 1. The second is that alternatives are employed in the derivation of both global and local effects. By contrast, while the ‘dual route’ approach being considered here also allows that an enrichment mechanism can be involved in items in both Experiments 3 and 1, this enrichment mechanism does not rely on alternatives. In addition, a second mechanism, which does rely on alternatives, only applies in the case of ‘global’ SIs, of the kind studied in Experiment 1.

There is little scope in this paper for a thorough empirical exploration of these two approaches.^7^ Here, we make two comments by way of comparison. First, the grammatical account could be integrated into a framework for reasoning with uncertainty since it implies a variety of interpretive possibilities for a sentence depending on whether the operator is inserted and where. Thus it is conceivable that the relation between the results of Experiment 1 and Experiment 3 above could be explained. However, that would require extra assumptions which link rates of insertion of the linguistic operator at the root level of a sentence (as would occur in Experiment 1) and in the scope of negation (as occurs in our Experiment 3).

Second, there is an important point of contrast between this grammatical account of our data and the one outlined in Bergen et al. (2016) and Potts et al. (2016). The latter approach proposes a simple narrowing mechanism to account for local enrichment, while the grammatical theory holds that upper-bound excluding local enrichments of expressions with scalar terms (compared with the many other kinds of local enrichment) are mediated by a syntactically represented exhaustification operator. Thus, the grammatical account would predict an effect of the distinctness of alternatives for local enrichments, comparable to that found for global enrichments. It is possible to investigate this prediction with our data. We can consider whether variation in ratings on our ‘so’ task (Experiment 3) are predicted by factors that are related to distinctness. To do this, we used a multiple regression analysis to test if semantic distance and boundedness significantly predicted participants’ ratings on the ‘so’ task. The results of the regression indicate that the two predictors did not significantly explain the variance [*R*^2^= 0.05, *F*(2,40) = 1.04, *p* = 0.36]. Neither semantic distance [β = -0.25, *t*(40) = -1.34, *p* = 0.19] nor boundedness [β = 0.23, *t*(40) = 0.84, *p* = 0.41] significantly predicted the ratings of ‘so’ task. Thus, a preliminary exploration of whether there is the predicted relationship between distinctness of alternatives and local enrichability was unable to find such a relation. This is unexpected if local enrichment relies on alternatives to the same extent as global. As mentioned, the RSA-LU approach assumes a general narrowing option for semantic interpretation as one of two routes to account for scalar enrichment, and this does not rely on alternatives.

To draw out the points of theoretical interest here, let us sum up what we have learnt from the scalar diversity effect. To date, previous studies (replicated here) have shown that factors relating to the distinctness of alternatives can explain some of Scalar Diversity, and this is predicted if SIs are derived by general Gricean reasoning or via a linguistically represented exhaustification operator. However, such factors explain by no means all of the scalar diversity effect. We outlined dual-route approaches above and showed that one version of that approach successfully explains more of the Scalar Diversity. Unlike the grammatical approach, RSA-LU suggests that mechanisms for deriving local enrichments do not rely on alternatives and thus the second source of potential variation, liability for UBELE, would be independent of factors such as the distinctness of alternatives. An analysis of results from Experiment 3 suggest this may be the case.

To turn to our final point of discussion, we point out that RSA-LU as stated does not shed much light on what factors might lead to the application of this ‘free enrichment’ mechanism used in achieving scalar effects. To put this another way, while the variability in local enrichment of the kind studied in Experiment 3 can partially explain variability in the inference task results, we are left with the question what explains the variability in the application of this second mechanism. For now, we have to leave this as a matter for future research.^8^ But, to re-iterate the point of discussion above, we learn from a comparison among theories which can account for local effects that a dual-route approach that does not rely on alternatives is better supported.

## Author Contributions

CS carried out the experiments and analyzed the data. CS and RB wrote the first draft of the manuscript. CS, YT, and RB have all contributed to the experimental design and the final version of the manuscript.

## Conflict of Interest Statement

The authors declare that the research was conducted in the absence of any commercial or financial relationships that could be construed as a potential conflict of interest.

## Footnotes

1 In this theoretical introduction, we set aside the widely discussed idea that both local ‘scalar’ enrichments of the kind in (1) and simple unembedded scalar enrichments are both derived via linguistic means in the form of a syntactically represented exhaustification operator (Fox, 2007; Chierchia et al., 2012). Interpreting our results in relation to this theory is different to dual route theories being discussed here. We will return to this point in greater depth below.

2 Some work on factors which impact on local enrichment include Chemla et al. (2017) and Sun and Breheny (2018c).

3 More details can be found in Bergen et al. (2016). See also Potts et al. (2016).

4 Bergen et al. (2016) consider two kinds of enrichments for scalar term W. One which excludes states of affairs that also support S (this is UBELE, mentioned in the text above) and one which includes only states of affairs where S is true. In our experiments below, we consider only the predictions of RSA-LU based on variation in the priors on the first kind of enrichment. In other work (Sun and Breheny, 2018b), we consider also the second kind of enrichment.

5 Our ‘so’ task is not the only way to get a measure of liability for UBELE. Previous research has used corpus methods to get a measure of this effect. See Chemla (2013) and Potts and Levy (2015).

6 We took data reported in van Tiel et al. (2016) P145 to build Figure 2 and ran comparative analysis in Experiments 2 and 3.

7 A more detailed empirically based comparison can be made on the basis of Sun and Breheny (2018b,c) but we leave this for future discussion.

8 In principle there are analytical possibilities as to how UBELE may arise without the use of alternatives. One possibility is that scalar terms (like, ‘warm’) may be upper-bounded in virtue of the presence of a maximality operation as suggested for the analysis of quantificational expressions with numerals (see Kennedy, 2015; Buccola and Spector, 2016). The research on numerals suggests there is a strong bias to understand those expressions via something like maximality (Geurts, 2006; Breheny, 2008). Other scalar expressions to which maximality may apply may simply vary in this bias.
