# Supplementary material for: A Link Between Local Enrichment and Scalar Diversity
Source: Front Psychol. 2018 Nov 1;9:2092. doi: 10.3389/fpsyg.2018.02092 (PMC6221952; doi:10.3389/fpsyg.2018.02092)
Supplement: Supplementary file 1 [file Data_Sheet_1.docx]

**Appendix**

1. Items used in Experiment 1

*Experimental sentences*

• adequate/good: The food | The salary is adequate. • allowed/obligatory: Copying | Drinking is allowed. • attractive/stunning: The singer | This model is attractive. • believe/know: The mother | The teacher believes it will happen. • big/enormous: That elephant | The house is big. • cheap/free: The food | The water is cheap. • content/happy: The homemaker | This child is content. • cool/cold: The air | The weather is cool. • dark/black: That fabric | The sky is dark. • difficult/impossible: The problem | The task is difficult. • dislike/loathe: The doctor dislikes coffee. The teacher dislikes fighting. • few/none: The biologist saw few of the birds. The cop saw few of the children. • funny/hilarious: This joke | This movie is funny. • good/perfect: The layout | This solution is good. • good/excellent: That movie | The food is good. • hard/unsolvable: The problem | The puzzle is hard. • hungry/starving: The boy | The dog is hungry. • intelligent/brilliant: The professor | This student is intelligent. • like/love: The actress likes the movie. The princess likes dancing. • low/depleted: The energy | The gas is low. • may/will: The teacher may come. This lawyer may appear in person. • may/have to: The boy may watch television. The child may eat an apple. • memorable/unforgettable: This movie | This party is memorable. • old/ancient: That house | That mirror is old. • palatable/delicious: The food | The wine is palatable. • participate/win: The runner | The skier participated. • possible/certain: Failing |Success is possible. • pretty/beautiful: The girl | The model is pretty. • rare/extinct: The plant | This bird is rare. • scarce/unavailable: This recording | This resource is scarce. • silly/ridiculous: That joke | That song is silly. • small/tiny: The car | This fish is small. • snug/tight: That dress | The shirt is snug. • some/all: The bartender saw some of the cars. The nurse saw some of the signs. • sometimes/always: The director is sometimes late. The doctor is sometimes irritable. • special/unique: That dress | That painting is special. • start/finish: The dancer | The runner started. • tired/exhausted: The runner | The worker is tired. • try/succeed: The athlete | The candidate tried. • ugly/hideous: That painting | The wallpaper is ugly. • unsettling/horrific: The movie | The news is unsettling. • warm/hot: The soup | The weather is warm. • wary/scared: The dog | The victim is wary.

*Control sentences*

• clean/dirty: The table is clean. • dangerous/harmless: The soldier is dangerous. • drunk/sober: The man is drunk. • sleepy/rich: The neighbor is sleepy. • tall/single: The gymnast is tall. • ugly/old: The doll is ugly. • wide/narrow: The street is wide.

1. Items used in Experiment 2

*Experimental sentences*

These were constructed on the following model: We took predicates from Experiment 1 to construct the materials on the model of ‘adequate/good’ below, unless this is otherwise specified below.

• adequate/good: The food | The salary is good but not adequate. • believe/know: The mother | The teacher knows it will happen but doesn’t believe it will happen. • dislike/loathe: The doctor loathes coffee but he does not dislikes coffee. The teacher loathes fighting but he does not dislike fighting. • few/none: The biologist saw none of the birds but it is not the case that he saw few of the birds. The cop saw none of the children but it is not the case that he saw few of the children. • like/love: The actress loves the move but she doesn’t like the movie. The princess loves dancing but she doesn't like dancing. • may/will: The lawyer will appear in person but it is not the case that he may appear in person. • may/have to: The boy has to watch television but it is not the case that he may watch television. The child has to eat an apple but it is not the case that he may eat an apple. • participate/win: The runner | The skier won but he did not participated. • some/all: The bartender saw all of the cars but not some of the cars. The nurse saw all of the signs but not some of the signs. • sometimes/always: The director is always late but he is not sometimes late. The doctor is always irritable but he is not sometimes irritable. • start/finish: The dancer | The runner finished but she did not start. • try/succeed: The athlete | The candidate succeed but he did not try.

*Control sentences*

The banker is rich but not happy. | The technology is sustainable but not affordable. | The assistant is busy but not effective. | The task is urgent but not important. | John left the party but he never came. | The woman has four children but not three children. | The man divorced his wife but he was never married.

1. Item used in Experiment 3

*Experimental sentences*

These were constructed on the following model: We took predicates from Experiment 1 to construct the materials on the model of ‘adequate/good’ below, unless this is otherwise specified below.

• adequate/good: The food | The salary is good so not adequate. • believe/know: The mother | The teacher knows it will happen so doesn’t believe it will happen. • dislike/loathe: The doctor loathes coffee so he does not dislikes coffee. The teacher loathes fighting so he does not dislike fighting. • few/none: The biologist saw none of the birds so it is not the case that he saw few of the birds. The cop saw none of the children so it is not the case that he saw few of the children. • like/love: The actress loves the move so she doesn’t like the movie. The princess loves dancing so she doesn't like dancing. • may/will: The lawyer will appear in person so it is not the case that he may appear in person. • may/have to: The boy has to watch television so it is not the case that he may watch television. The child has to eat an apple so it is not the case that he may eat an apple. • participate/win: The runner | The skier won so he did not participated. • some/all: The bartender saw all of the cars so not some of the cars. The nurse saw all of the signs so not some of the signs. • sometimes/always: The director is always late so he is not sometimes late. The doctor is always irritable so he is not sometimes irritable. • start/finish: The dancer | The runner finished so she did not start. • try/succeed: The athlete | The candidate succeed so he did not try.

*Control sentences*

The woman has four children so not three children. | The window is open so not closed. | The cup is red so not blue. | John left the party so he never came. | The train arrived so it never departed. | The man divorced his wife so he was never married. | The banker is rich so not happy.
